# Endovascular embolization versus surgical clipping in a single surgeon series of basilar artery aneurysms: a complementary approach in the endovascular era

**DOI:** 10.1007/s00701-021-04803-5

**Published:** 2021-03-10

**Authors:** Ethan A. Winkler, Anthony Lee, John K. Yue, Kunal P. Raygor, W. Caleb Rutledge, Roberto R. Rubio, S. Andrew Josephson, Mitchel S. Berger, Daniel M. S. Raper, Adib A. Abla

**Affiliations:** 1 Department of Neurological Surgery, University of California, San Francisco, CA USA; 2 Department of Neurology, Weill Institute for Neurosciences, University of California, San Francisco, CA USA

**Keywords:** Aneurysm, Endovascular coil embolization, Microsurgical clipping, Outcomes

## Abstract

**Background:**

Currently, most basilar artery aneurysms (BAAs) are treated endovascularly. Surgery remains an appropriate therapy for a subset of all intracranial aneurysms. Whether open microsurgery would be required or utilized, and to what extent, for BAAs treated by a surgeon who performs both endovascular and open procedures has not been reported.

**Methods:**

Retrospective analysis of prospectively maintained, single-surgeon series of BAAs treated with endovascular or open surgery from the first 5 years of practice.

**Results:**

Forty-two procedures were performed in 34 patients to treat BAAs—including aneurysms arising from basilar artery apex, trunk, and perforators. Unruptured BAAs accounted for 35/42 cases (83.3%), and the mean aneurysm diameter was 8.4 ± 5.4 mm. Endovascular coiling—including stent-assisted coiling—accounted for 26/42 (61.9%) treatments and led to complete obliteration in 76.9% of cases. Four patients in the endovascular cohort required re-treatment. Surgical clip reconstruction accounted for 16/42 (38.1%) treatments and led to complete obliteration in 88.5% of cases. Good neurologic outcome (mRS ≤ 2) was achieved in 88.5% and 75.0% of patients in endovascular and open surgical cohorts, respectively (*p* = 0.40). Univariate logistic regression analysis demonstrated that advanced age (OR 1.11[95% CI 1.01–1.23]) or peri-procedural adverse event (OR 85.0 [95% CI 6.5–118.9]), but not treatment modality (OR 0.39[95% CI 0.08–2.04]), was the predictor of poor neurologic outcome.

**Conclusions:**

Complementary implementation of both endovascular and open surgery facilitates individualized treatment planning of BAAs. By leveraging strengths of both techniques, equivalent clinical outcomes and technical proficiency may be achieved with both modalities.

## Introduction

Basilar artery aneurysms (BAAs) can be challenging to treat. Randomized controlled trials have reported superior outcomes with endovascular coiling for ruptured aneurysms compared to surgical clipping, most noticeably in the posterior circulation [28, 40, 46]. Extrapolation of randomized trial results to all aneurysms has led to increased use of endovascular techniques over time. Currently, most centers favor treating basilar artery aneurysms endovascularly. A subset of aneurysms, however, are not safely treated with existing endovascular options—such as those with complex and/or wide neck configurations, perforator or blister-like morphology, or eloquent vessels arising from the dome [38, 39].

When treating BAAs, endovascular options and surgery are therefore complementary rather than competing techniques. Expertise with both modalities enables tailoring of treatment strategy to individual patients, but trade-offs exist between the minimal invasiveness of endovascular and the durability of surgical therapy. To develop comprehensively trained cerebrovascular neurosurgeons, organized neurosurgery has made efforts to incorporate neuroendovascular skills into neurosurgical training [12]. Despite initial skepticism, studies have supported that the same practitioners may acquire sufficient expertise to perform both open and endovascular neurosurgery safely [3, 4, 8].

Early career dual-trained neurosurgeons are more likely to treat cerebral aneurysms with neurointerventional techniques [16]. With continued evolution of endovascular technologies, the proportion of intracranial aneurysms treated with open surgery continues to decline. This results in reduced exposure for neurosurgical residents and now commonly necessitates post-graduate fellowships in open vascular neurosurgery for sufficient exposure to complex open cases [9, 22]. In the current era of cerebrovascular surgery, the assumption may exist that a neurosurgeon with the training to perform both surgical and endovascular treatment modalities would almost exclusively treat BAAs with endovascular techniques. Whether open microsurgery would be utilized or required, and to what extent, for treating BAAs in a series of patients treated by a surgeon who performs both treatment modalities is not presently known.

Here, we describe a single surgeon case series of 42 consecutively treated BAAs, including those arising from the basilar artery apex, trunk, or perforators, with either endovascular coiling or microsurgical clip reconstruction within the first 5 years of clinical practice. We describe our philosophy of patient selection, leveraging the strengths of both open and endovascular surgery, and demonstrate similar outcomes compared with clinical series of basilar aneurysms treated by a single approach.

## Methods

### Patient population, inclusion and exclusion criteria

A prospectively maintained database of all patients with aneurysms treated from 2014 to 2019 was reviewed to identify adult patients undergoing endovascular or open surgical treatment for ruptured or unruptured BAAs. Aneurysms included in this analysis arose from the basilar artery apex, basilar artery trunk, or basilar artery perforators. Aneurysms arising from the anterior inferior cerebellar, posterior inferior cerebellar, superior cerebellar, or vertebral arteries were excluded. All endovascular coiling and open microsurgical clipping procedures were performed by the senior author (AAA). Institutional Review Board approval was obtained prior to data collection, and consent was obtained prospectively for each patient included in the series.

### Clinical decision-making

Each patient underwent pre-intervention computed tomography angiography (CTA) or digital subtraction angiography (DSA). In cases when CTA allowed sufficient visualization of the aneurysm morphology, aneurysm neck, and provided adequate 3d reconstruction, DSA was not pursued given some minor additional risk involved to the patient. Patients were evaluated and discussed by a multi-disciplinary team comprised of a dual-trained vascular neurosurgeon, interventional neuroradiologists, and neurovascular neurologists. The default treatment modality was endovascular therapy. After multi-disciplinary discussion and evaluation, the treatment option was determined by the treating surgeon, in consultation with the patient. Ultimately, assignment of treatment modality incorporated aneurysm and patient characteristics: aneurysm size and morphology, patient demographics, patient-specific psychosocial factors, and the perceived safety and durability for each treatment modality (see *Discussion*). Surgical treatment was employed in cases in which an endovascular approach was deemed high risk for complication, impossible to complete without vessel/perforator sacrifice, unlikely to secure the aneurysm (most notably for ruptured aneurysms), or when it would necessitate the use of a stent and consequently also dual antiplatelet therapy in the setting of subarachnoid hemorrhage.

### Endovascular therapy

**Fig. 1:**
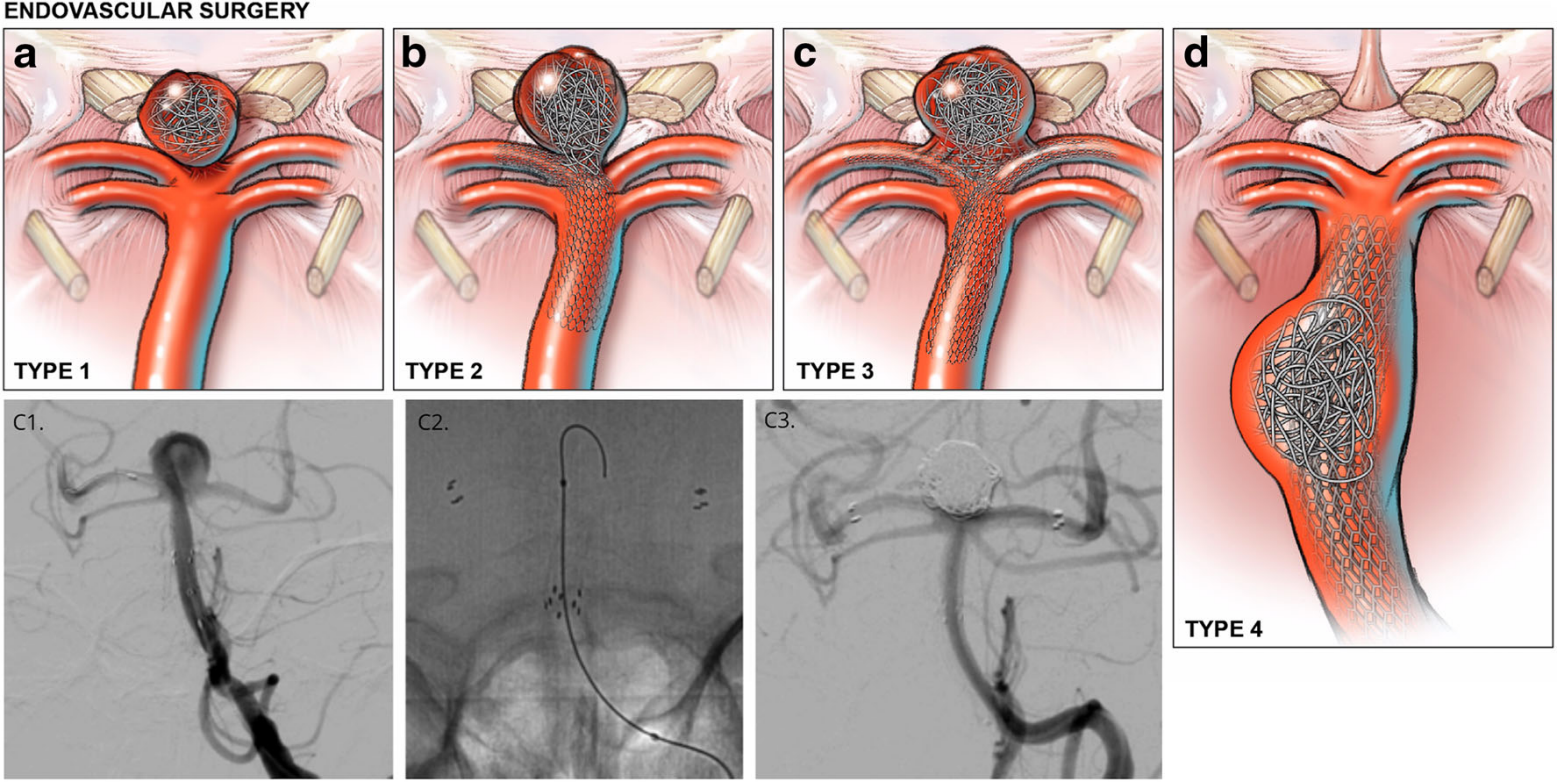
Representative illustrations of endovascular interventions for basilar artery aneurysms. (**a**–**c**) For basilar tip aneurysms, the relationship between the aneurysm neck and origin of the posterior cerebral artery (PCA) influences intervention selection. (**a**) Narrow-necked aneurysms with no involvement of proximal P1 origins are primarily coiled. (**b**–**c**) Wide-necked aneurysms involving one or both P1 origins are treated with a single stent or two stents in Y-configuration, respectively, to facilitate coil embolization. Inset (C1, C2, C3), representative intraprocedural angiography demonstrating optimal stent positioning and coil embolization resulting in complete aneurysm obliteration of a wide neck basilar artery apex aneurysm (Type 3). Injection, left vertebral. Projection, anterior-posterior. (**d**) For select aneurysms of the basilar trunk with the ability to circumferentially coil embolize a saccular component, a microcatheter is jailed with a stent in the basilar trunk to maintain luminal patency. Stent-assisted coiling is then performed

Endovascular treatments were performed under general anesthesia through transfemoral arterial access. For unruptured aneurysms, following femoral artery access, an initial 70-U/kg heparin bolus was given and activated clotting time confirmed to be at least 2.5× that of the patient’s baseline. For ruptured aneurysms, half the heparin bolus was given initially with the remaining half of the heparin bolus given after placement of the first coil. All coil embolization was performed with bare platinum coils. When a stent was anticipated, patients were pretreated with aspirin 325 mg and clopidogrel 75 mg for 10 to 14 days prior to the procedure. Aspirin response assays and Plavix response assays using the ARU and PRU values were performed to ensure adequate levels of platelet inhibition had been achieved (Verify Now). For clopidogrel non-responders, patients were switched from clopidogrel to prasugrel (Effient), after administration of a loading dose of 60 mg. The decision to utilize a stent was based on (1) the rupture status of the patient and (2) the neck diameter and relationship with bilateral P1 segments of the posterior cerebral arteries (PCAs) (Fig. 1). We avoid use of an intracranial stent in ruptured aneurysm patients due to reported morbidity associated with dual anti-platelet therapy. For wide-necked aneurysms, one or two Neuroform stents were placed (Stryker Neurovascular, Fremont, CA). For those wide-necked aneurysms incorporating a single P1 in the wide neck, a single stent was placed to span from the PCA vessel that was incorporated into the aneurysm neck into the distal basilar artery. For aneurysms with a neck that incorporates the bilateral P1 segments, two stents were placed in Y-configuration in both PCAs into the distal basilar artery (Fig. 1). For ruptured aneurysms or aneurysms in which primary unassisted coiling resulted in herniation of coils into the parent vessel but did not incorporate the P1 segments, either balloon-assisted coiling or utilization of a two-microcatheter technique was performed to further stabilize the framing coil. All patients undergoing stent-assisted coiling were maintained on dual anti-platelet therapy for a minimum of 6 months. Following the first treated BAA patient/aneurysm through an endovascular intervention, which resulted in a thrombo-embolic complication approximately 8 h after the procedure, all subsequent unruptured endovascularly treated aneurysm patients were placed on an infusion of intravenous heparin overnight (after computed tomography (CT) brain confirmed no hemorrhage) with a PTT goal of 50 to 60 until the following morning.

### Open microsurgical clip reconstruction

**Fig. 2:**
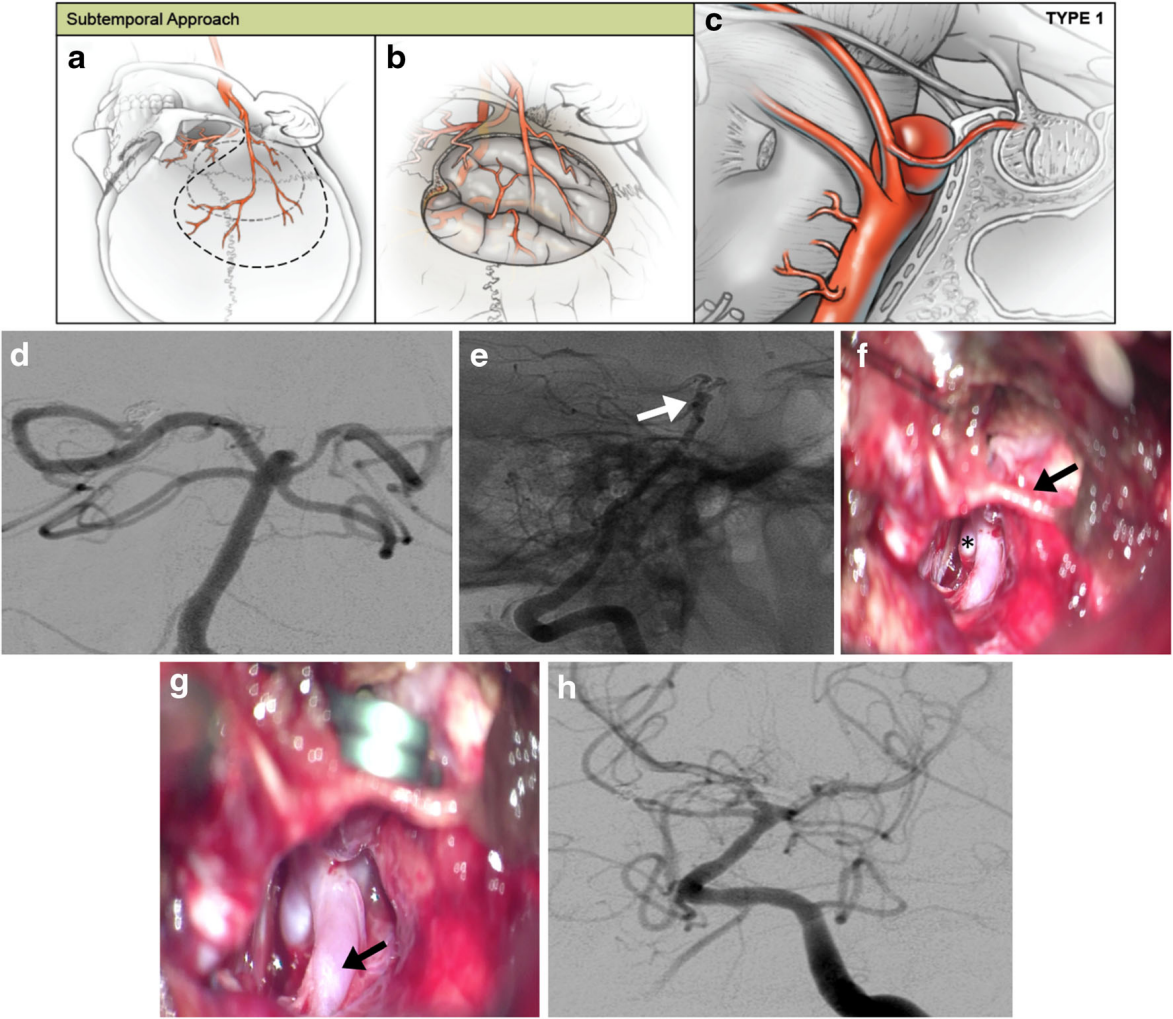
Representative subtemporal approach to basilar artery aneurysms. (**a**–**c**) For low-lying basilar apex aneurysms located inferior to the dorsum sella, a subtemporal approach offers direct visualization of the aneurysm neck, whereas a standard orbitozygomatic pterional craniotomy is limited by the dorsum. (**a**, **b**) A horseshoe incision is flapped inferiorly, and a temporal craniotomy is performed with further care to use the high-speed drill to flatten the temporal bone adjacent to the middle fossa floor. (**c**) The temporal lobe is retracted away from the middle fossa floor, and the basilar artery is visualized. The posterior communicating artery can be sectioned at the P1-P2 junction to aid in the medial mobilization of the ICA although this is rarely necessary. (**d**–**g**) Representative images of a low-lying posteriorly projecting ruptured basilar apex aneurysm that had already undergone unsuccessful attempted coiling. (**d**, **e**) Preoperative AP and lateral angiograms with white arrow representing a posteriorly projecting basilar apex aneurysm that is unsuitable for orbitozygomatic pterional Trans-Sylvian approach. (**f**) Intraoperative photograph demonstrates aneurysm dome (asterisk) and fourth cranial nerve (arrow). (**g**) Intraoperative photograph demonstrates temporary clip on the proximal basilar trunk. The P1 segment of the ipsilateral PCA is indicated (arrow). (**h**) Immediate postoperative AP digital subtraction angiogram demonstrates complete angiographic occlusion of the aneurysm

**Fig. 3:**
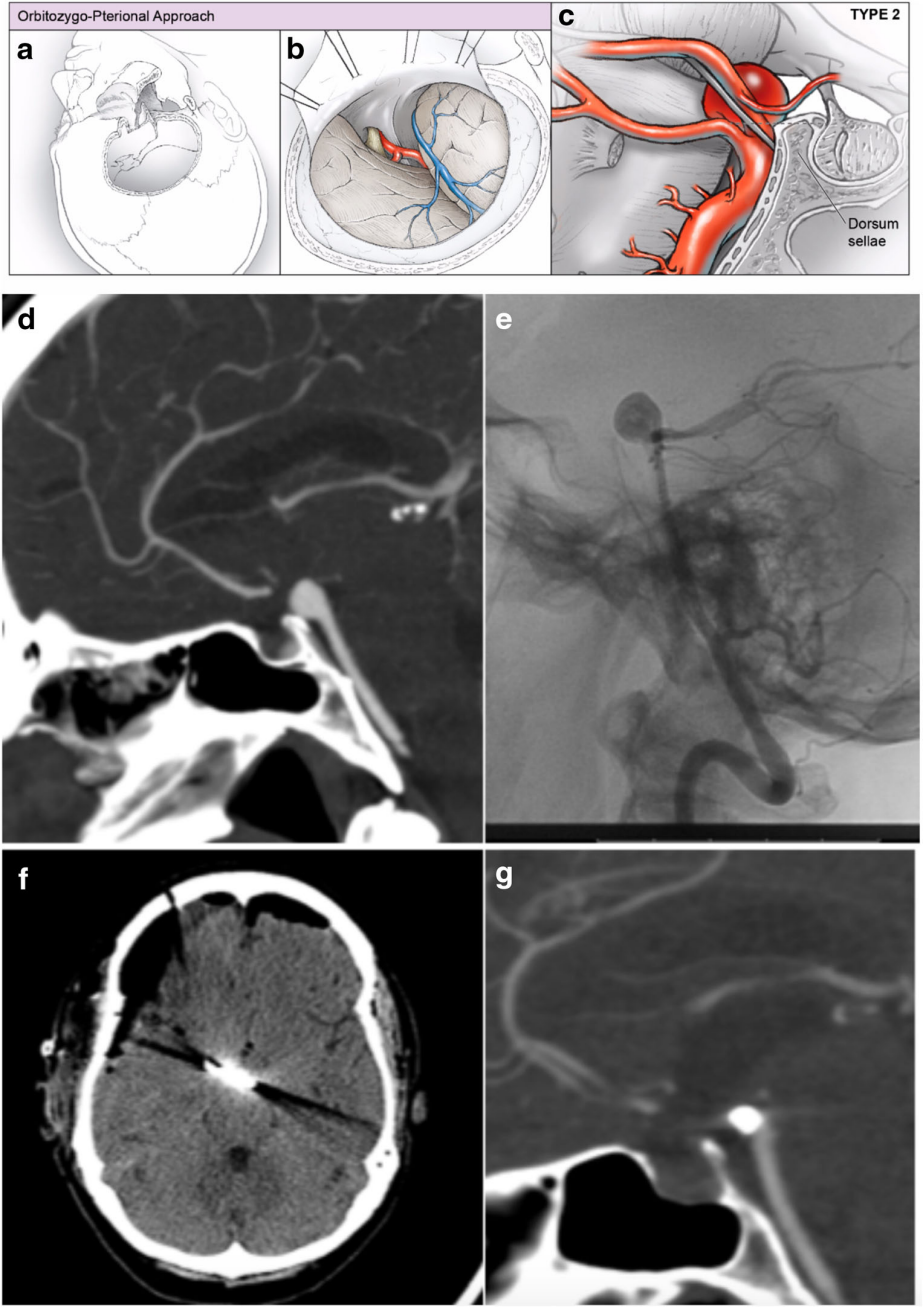
Representative orbitozygomatic-pterional approach to basilar artery aneurysms. (**a**–**c**) For basilar apex aneurysms located at or above the level of the dorsum sella, an orbitozygomatic-pterional approach offers visualization of the aneurysm neck. (**a**, **b**) After a standard curvilinear incision, a pterional craniotomy with orbitozygomatic osteotomies is performed. (**c**) Trans-Sylvian dissection is used to expose the basilar artery apex. The posterior communicating artery can be sectioned at the P1-P2 junction to aid in the medial mobilization of the ICA. (**d**–**g**) Representative images of management of a basilar apex aneurysm. (**d**, **e**) Preoperative sagittal CT angiogram and lateral angiograms demonstrate anterior and superiorly projecting aneurysm. (**f**) Immediate postoperative axial non-contrast CT scan and (**g**) 2-year follow-up CT angiogram demonstrate complete occlusion of the aneurysm

**Fig. 4:**
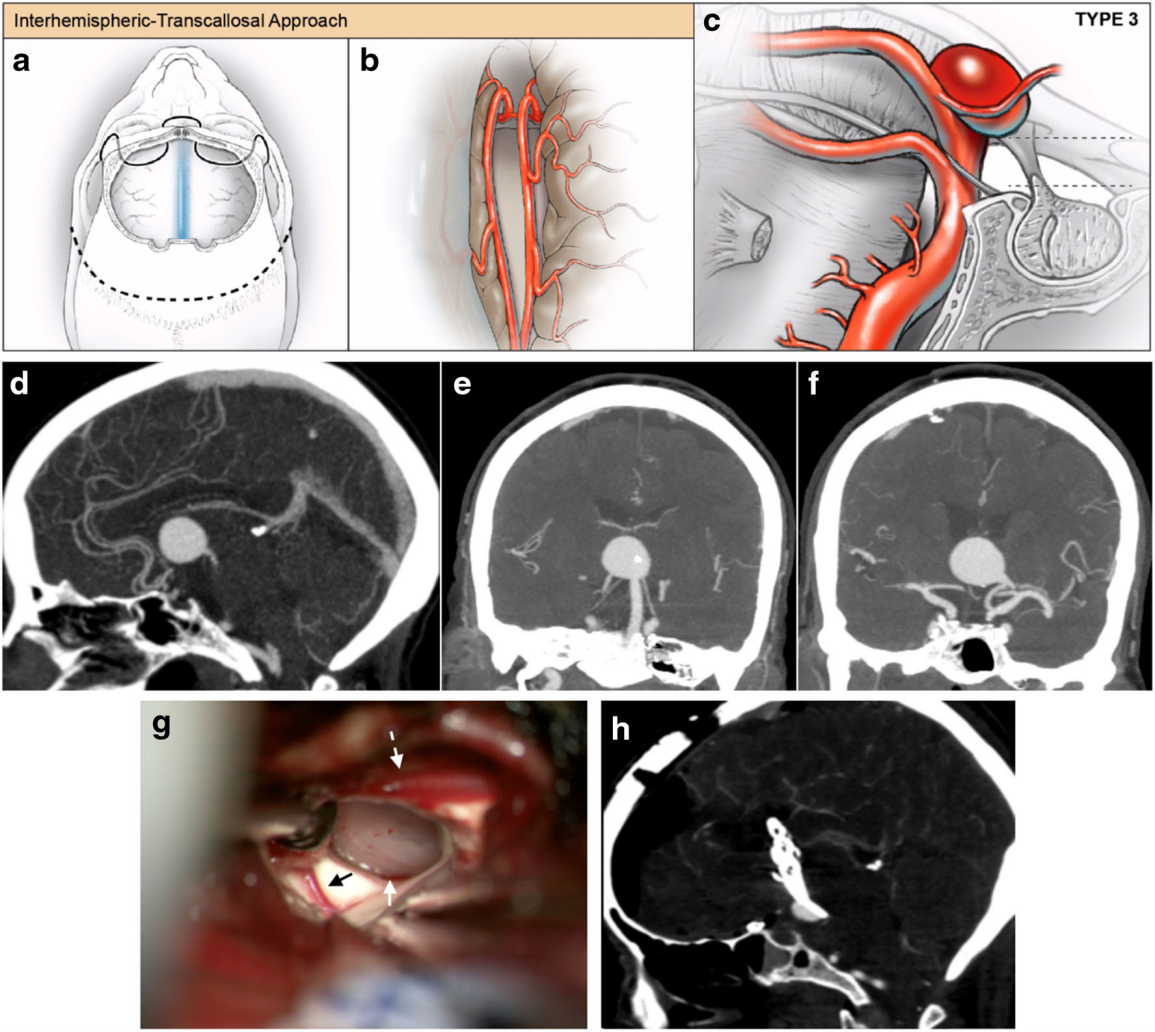
Representative interhemispheric-transcallosal approach to basilar artery aneurysms. (**a**–**c**) For basilar apex aneurysms that are significantly superior to the dorsum sella and are projecting into or located completely within the third ventricle, an interhemispheric-transcallosal approach offers visualization of the aneurysm neck. This was performed for one patient in this case series. (**a**) After a standard bicoronal incision, a bifrontal craniotomy is performed. (**b**) Interhemispheric dissection is performed to dissect the pericallosal arteries away from the corpus callosum, and the corpus callosum is opened and the right lateral ventricle is entered first. To widen the surgical corridor into the third ventricle, the opening into the third ventricle through the foramen of Monroe is further extended posteriorly by performing a trans-choroidal fissure approach. Simultaneously, a pterional approach was performed for proximal control of the basilar artery trunk via a trans-Sylvian corridor which allowed temporary clipping of the basilar artery and aneurysm softening. (**d**–**h**) Representative images of management of an unruptured giant partially fusiform basilar apex aneurysm. (**d**–**f**) Preoperative sagittal (**d**) and coronal (**e**, **f**) CT angiogram demonstrates anterior and superiorly projecting aneurysm into the third ventricle. (**e**, **f**) Coronal images demonstrate the fusiform nature of the aneurysm with both PCAs originating from the aneurysm dome. (**g**) Intraoperative photograph demonstrates view through the Foramen of Monroe with aneurysm dome visible (white arrow). The thalamostriate vein (black arrow) and anterior septal vein (dashed white arrow) are visible. The aneurysm is dissected free from ependymal attachments to visualize the neck and clip the aneurysm. (**h**) Immediate postoperative sagittal CT angiogram demonstrates a base remnant of the fusiform aneurysm from which the posterior cerebral arteries exit

For open microsurgical clip reconstruction, a frontotemporal-orbitozygomatic (FT-OZ), subtemporal, transcallosal, or retrosigmoid craniotomy was performed. Approach selection was driven by the relationship of the aneurysm with the dorsum sellae (Figs. 2, 3, and 4). For aneurysms of the basilar apex, the orbitozygomatic approach was utilized for aneurysms above or at the level of the dorsum sella. For basilar artery apex aneurysms occurring in a retro-sellar location or those with complete posterior projection of the aneurysm dome, a subtemporal trans-tentorial approach was utilized, with skeletonization of cranial nerve IV and sectioning of the tentorium between the 3rd and 5th cranial nerves.

For aneurysms arising from basilar artery perforators or the basilar artery trunk, approach selection was determined by the relationship of the aneurysm (as well as the portion of the basilar artery needed for proximal control) with cranial nerves (CN) V and VII as follows: access above CN V, subtemporal-trans-tentorial approach; access required between CN V and CN VII, subtemporal transtentorial approach with anterior petrosectomy; and access required inferior to CN VII, extended retrosigmoid approach. All operations were conducted with neurophysiologic monitoring, including motor evoked and somatosensory evoked potentials. All patients were confirmed to be in burst suppression prior to temporary clip application.

Temporary cardiac standstill with use of intravenous administration of adenosine was utilized to soften the aneurysm dome in cases when proximal control was available but required temporary clipping in a limited window that placed risk to cranial nerves or those cases in which the aneurysm dome remained tense with temporary clipping. Permanent clips were applied to completely occlude the aneurysm while maintaining patency of the parent artery and adjacent perforators. Aneurysm occlusion and perforator patency in proximity to the aneurysm neck and aneurysm clips were confirmed with visual inspection, micro-Doppler ultrasonography, and indocyanine green videoangiography.

### Data collection and statistical analyses

Clinical and radiographic variables were retrospectively collected through the electronic medical record. For endovascular cases, aneurysm occlusion was classified by the Raymond Roy Occlusion Classification (RROC) [7], and either RROC 1 or RROC 2 was classified as complete occlusion as previously described [5, 43]. An analogous grading scale was applied to open surgical clipping (complete, small neck remnant, or >5% of the original aneurysm remaining) as we have previously described [43]. Aneurysm occlusion was independently adjudicated by an endovascular fellowship-trained neurosurgeon on postoperative DSA or high resolution CTA. Cerebrovascular infarction was clinically defined by a focal neurologic deficit in a territory of a major cerebral artery which persisted for > 24 h, and when available, confirmed on magnetic resonance imaging (MRI). Neurological outcome was assessed and dichotomized by using the modified Rankin score (mRS) (good outcome, mRS 0–2; poor outcome, mRS 3–5) [36, 45, 47].

Data are presented as means and standard deviations (SD) or proportions, for continuous and categorical variables, respectively. Pearson’s chi-squared test (*X*^*2*^) and analysis of variance (ANOVA) were used to assess differences between ruptured and unruptured cohorts. Univariate logistic regression was performed to identify predictors of aneurysm occlusion or neurologic outcome. Statistical significance was assessed at *p*<0.05.

## Results

### Patient demographic information and clinical characteristics

From December 2014 to September 2019, 42 endovascular and open microsurgical procedures were performed in 34 patients to treat BAAs. Patient demographic information is summarized in Table 1. Treated aneurysms arose from the basilar artery apex, trunk, or trunk perforators in 78.6%, 16.6%, 4.8% of cases, respectively, and include 10 aneurysms with fusiform morphology (23.8 %). Unruptured BAAs accounted for 35 of 42 cases (83.3%). The BAA represented the rupture site in all cases of subarachnoid hemorrhage.

**Table 1:** Patient demographics and outcome data for patients with basilar artery aneurysms treated with endovascular coiling or open microsurgical clip ligation

	Treatment		*p*-value
	Coil (*n*=26)	Clip (*n*=16)	
Sex, *n* (%)			0.658
Male	3 (11.5%)	3 (18.8%)	
Female	23 (88.5%)	13 (81.2%)	
Age in years, mean (SD)	60.0 (9.8)	54.3 (12.5)	0.132
Subarachnoid hemorrhage, *n* (%)			0.999
Yes	4 (15.4%)	3 (18.8%)	
No	22 (84.6%)	13 (81.3%)	
Aneurysm dome size in mm, mean (SD)	8.2 (4.4)	8.7 (7.0)	0.813
Aneurysm neck size in mm, mean (SD)	5.2 (3.1)	6.6 (6.5)	0.441
Aneurysm dome to neck ratio, mean (SD)	1.7 (0.5)	1.4 (0.5)	0.068
Aneurysm location, *n* (%)			0.104
Apex	20 (76.9%)	13 (81.3%)	
Trunk	6 (23.1%)	1 (6.2%)	
Perforator	0 (0.0%)	2 (12.5%)	
Morphology, *n* (%)			0.464
Fusiform	5 (19.2%)	5 (31.2%)	
Saccular	21 (80.8%)	11 (68.8%)	
VP shunt placement, *n* (%)			0.999
Yes	4 (15.4%)	2 (12.5%)	
No	22 (84.6%)	14 (81.5%)	
Length of stay in days, mean (SD)	5.5 (8.2)	10.0 (8.7)	0.115
Any adverse event, *n* (%)			0.658
Yes	3 (11.5%)	3 (18.8%)	
No	23 (88.5%)	13 (81.2%)	
Discharge site, n (%)			0.001*
Home	24 (92.4%)	8 (50.0%)	
Acute rehab/SNF	1 (3.8%)	7 (43.8%)	
Death	1 (3.8%)	1 (6.2%)	
Follow-up duration, days (SD)	380.7 (224.2)	353.1 (290.4)	0.747
Aneurysm obliteration at last follow-up, *n* (%)			0.68
Yes	20 (76.9%)	14 (87.5%)	
No	6 (23.1%)	2 (12.5%)	
mRS at last follow-up, *n* (%)			0.397
0–2	23 (88.5%)	12 (75.0%)	
3–6	3 (11.5%)	4 (25.0%)	

Endovascular coiling was the default treatment (Fig. 1) and accounted for 26 of 42 treatments (61.9%), while microsurgical clip reconstruction accounted for 16 of 42 treatments (38.1%). Endovascular treatment included 17 (65.4%) stent-assisted coiling procedures and 9 (34.6%) non-stent-assisted coiling procedures. No flow-diverting stents or intrasaccular devices were utilized over the study period. For stent-assisted coiling, a single stent was placed in in 11 cases (64.7%) and two stents were placed in y-configuration with distal purchase in bilateral P1 segments of the PCA in 6 cases (35.3%) (Fig. 1). Dual antiplatelet therapy was utilized in all patients undergoing stent-assisted coiling—including aspirin/clopidogrel and aspirin/prasugrel in 16 and 1 patient(s), respectively. No stent-assisted coiling was performed in any ruptured aneurysm.

All patients in the open microsurgery group underwent clip reconstruction. Indications for open surgery included unfavorable branch vessel anatomy for endovascular therapy (6/16, 37.5%), younger patient with preference for fewer treatments (3/16, 18.8%), basilar artery perforator aneurysms (3/16, 18.8%), poor compliance or intolerance of dual antiplatelet therapy (2/16, 12.5%), wide-necked basilar apex aneurysm in the rupture setting after failed coiling (1/16, 6.2%), or other psychosocial factors precluding clinical follow-up (1/16, 6.2%). In the open surgical cohort, one patient was lost to follow-up for over 1 year, continued to use methamphetamines, and later required a second open surgical procedure due to regrowth of residual fusiform basilar apex aneurysm. The case was aborted and immediately crossed over for stent-assisted endovascular therapy, given the inability to visualize the perforators, surrounding cranial nerves, and aneurysm neck, all due to subarachnoid scarring after the prior surgery.

In the open surgical cohort, approach selection was largely driven by the relationship of the aneurysm with the dorsum sella—including 9 (56.2%) frontotemporal-orbitozygomatic (FT-OZ), 5 (31.2%) subtemporal, 1 (6.2%) transcallosal, and 1 (6.2%) retrosigmoid approaches (Figs. 2, 3, and 4). For FT-OZ or subtemporal approaches, the posterior communicating artery was divided adjacent to the P1-P2 junction (away from any posterior communicating artery perforators) in 5 (35.7%) of 14 cases to widen the operative corridor between the internal carotid artery and the posterior cerebral artery. Temporary clipping was used in 9 (56.2%) cases to facilitate aneurysm dissection, and in cases of intraoperative rupture, which occurred in 2 (12.5%) of 16 cases. Adenosine was used to induce temporary cardiac arrest in 6 (37.5%) cases and was successful in obtaining asystole in all cases utilized.

No statistically significant differences were observed in patient or aneurysm characteristics between cohorts (Table 1). Mean diameter of treated aneurysms was 8.4 ± 5.5 mm. A statistical trend was observed towards smaller dome-to-neck ratios in aneurysms treated with microsurgical clip reconstruction (*p* =0.07).

### Aneurysm obliteration and predictors of complete occlusion

Rates of complete aneurysm occlusion were 76.9% and 87.5% for endovascular and open surgery, respectively. In the endovascular cohort, 4 patients required re-treatment (range: 1–2 procedures). One patient with SAH was intentionally brought back for delayed stenting after the rupture site was secured with coiling on initial presentation. The other three patients underwent repeat treatment as the result of coil compaction or growth of a residual aneurysm. Within the endovascular cohort, stent-assisted coiling was associated with higher rates of occlusion than non-stent-assisted coiling, but failed to reach statistical significance (stent-assisted coiling, 82.4%; non-stent-assisted coiling, 66.7%, *p* = 0.63). Five of 6 (83.3%) endovascular treated cases and two of two (100%) surgically treated cases with incomplete obliterations occurred with fusiform aneurysm morphology. Univariate logistic regression analysis identified younger age (OR 1.23 [95% CI 1.03–1.48]), increasing dome diameter (OR 0.72 [95% CI 0.55–0.95]), increasing aneurysm neck diameter (OR 0.56 [95% CI 0.36–0.89]), and fusiform morphology (*p* <0.05) as predictors of incomplete occlusion.

### Clinical outcomes

**Fig. 5:**
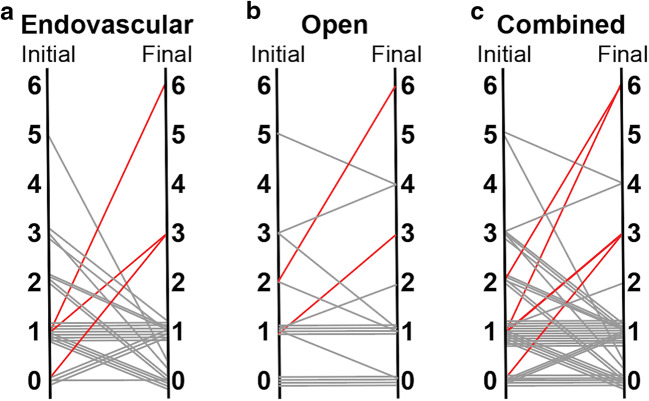
Clinical outcomes following endovascular and open surgical treatment of basilar artery aneurysms. (**a**–**c**) Graph depicting modified Rankin score (mRS) following endovascular, open and combined treatment cohorts. Left axis, pre-intervention mRS. Right axis, mRS at last follow-up. Red, clinically significant morbidity or mortality. Gray, unchanged or improved disability

Mean clinical follow-up was 370 ± 248 days. Good neurologic outcome (mRS ≤ 2) was obtained in 35 of 42 (83.3%) cases (endovascular: 88.5%, open surgery: 75.0%, *p* =0.40). Patients neurologically improved or remained unchanged following 37 of 42 procedures (rate of permanent neurologic morbidity: 11.9%). Rates of permanent neurologic morbidity for endovascular and open surgery were 11.5% and 12.5%, respectively (Fig. 5).

### Procedure-related adverse events

In the endovascular cohort, there were three procedure-related adverse events—including 1 gastrointestinal bleed in a patient on dual antiplatelet therapy, 1 thalamic stroke and contralateral SCA stroke (in the same patient), and 1 iatrogenic subarachnoid hemorrhage. The patient with iatrogenic SAH was found to have subarachnoid hemorrhage on the routine postoperative CT scan during delayed retreatment of her previously ruptured basilar artery aneurysm, but there was no evidence of neurologic change or intraprocedural contrast extravasation. She made a full recovery and was discharged without resultant. The thalamic/SCA stroke occurred in delayed fashion 8 h after a non-stent-assisted coil embolization procedure of a wide-neck aneurysm. This was the first endovascular patient treated and the patient initially woke up neurologically intact but suffered thalamic strokes as well as a contralateral SCA stroke, presumably after the heparin’s effect had worn off. The patient initially recovered from the infarcts but suffered a complication following percutaneous gastrostomy placement resulting in sepsis, after which the family withdrew any further supportive care. Following this event, in subsequent endovascularly treated coil embolization of basilar artery aneurysms, all patients were kept on heparin infusion overnight (for unruptured aneurysms) until the following morning.

In the open surgery cohort, there were three procedure-related adverse events, including 1 bi-thalamic stroke, one death from delayed hemorrhage, and 1 pontine stroke. The rate of ischemic stroke across modalities was 7.1% (by modality, endovascular: 3.8%, open surgery: 12.5%, *p* = 0.55). There was a single mortality in each cohort (mortality rate: endovascular, 3.8%; open surgery, 6.2%, *p* = 0.99). The open surgical treatment involved a giant fusiform aneurysm of the basilar apex located completely within the third ventricle which presented with hydrocephalus and lethargy. The patient did not have sufficient posterior communicating (PCOM) artery collateral to tolerate basilar occlusion, and angiography demonstrated the P1 vessels arose directly from the side of the aneurysm preventing catheterization and Y-configuration stent coiling. Both flow diversion and bypass options were considered for this difficult giant partially fusiform basilar apex aneurysm. Flow diversion was not elected as an option here given the perceived risk of placing a flow diversion construct across perforators of the basilar trunk and basilar apex. Bypass (and basilar occlusion) was not elected for this case because the aneurysm incorporated the P1 segments of the PCA and bypass to the PCA would have needed to be done bilaterally and also still resulted in filling of the aneurysm through the bypass retrograde from P1 (assuming basilar occlusion performed below SCA). Following ventriculostomy placement, the patient was treated with a pterional approach with orbitotomy for proximal basilar artery control and a transcallosal approach for clipping of the aneurysm through a trans-choroidal fissure trans-foramen of Monro corridor. The patient initially was unchanged neurologically, but her aneurysm could not be completely eliminated surgically given the fusiform nature and exit of the P1 segments directly from the lateral sides of the aneurysm dome. The patient re-ruptured approximately 1 week after surgery from the aneurysm remnant and immediately progressed to brain death with bilateral fixed and dilated pupils.

Univariate logistic regression analysis was performed to identify predictors across treatment modalities. This analysis identified advancing patient age (OR 1.11 [95% CI 1.01–1.23]) and procedure-related adverse event (OR 85.0 [95% CI 6.5–1118.9]) as predictors of poor neurologic outcome with treatment of BAAs. Treatment modality (endovascular coiling or surgical clipping) was not associated with neurologic outcome (OR 0.39 [95% CI 0.08–2.04]) (Table 2).

**Table 2:** Predictors of poor neurologic outcome in combined endovascular and open surgical treatment of basilar artery aneurysms

	Good outcome (mRS 0–2)	Poor outcome (mRS 3–6)	OR (95% CI)	*p*-value
Sex, *n* (%)			NA	0.567
Male	6	0		
Female	29	7		
Age in years, mean (SD)	56.1 (10.7)	66.6 (9.5)	1.11 (1.01–1.23)	0.028*
Subarachnoid hemorrhage, *n* (%)			0.17 (0.03–1.07)	0.077
Yes	4	3		
No	31	4		
Aneurysm dome size in mm, mean (SD)	8.5 (5.4)	8.1 (6.4)	0.99 (0.84–1.15)	0.871
Aneurysm neck size in mm, mean (SD)	6.0 (5.0)	4.3 (2.0)	0.84 (0.58–1.21)	0.378
Aneurysm dome to neck ratio, mean (SD)	1.5 (0.4)	1.7 (0.8)	1.93 (0.45–8.24)	0.568
Aneurysm location, n (%)			0.57 (0.01–5.91)	0.999
Apex	27	6		
Trunk/perforator	8	1		
Morphology, *n* (%)			2.04 (0.20–105.94)	0.999
Fusiform	9	1		
Saccular	26	6		
Any adverse event, *n* (%)			85.0 (6.5–1118.9)	<0.001*
Yes	1	5		
No	34	2		
Follow-up duration, days (SD)	355.6 (226.7)	443.1 (350.5)	1.00 (1.00–1.01)	0.546
Aneurysm obliteration at last follow-up, *n* (%)			0.89 (0.13–7.14)	0.999
Yes	21	4		
No	14	3		
Intervention, *n* (%)			0.39 (0.08–2.04)	0.397
Clip	12	4		
Coil	23	3		
Preoperative mRS, *n* (%)			5.81 (0.94–36.00)	0.077
0–2	31	4		
3–6	4	3		

## Discussion

In this report, we review our approach and outcomes with endovascular or open treatment of aneurysms arising from the basilar artery—including those arising from the basilar artery apex, trunk, or perforators. To our knowledge, this is the largest single surgeon series utilizing both endovascular and open treatment of BAAs in the literature. Our data supports the conclusion that technical proficiency, and more importantly favorable clinical outcomes, may be obtained relatively early in one’s career through thoughtful patient selection, leveraging the strengths of each treatment modality.

### Patient selection: endovascular coiling vs. open surgical clipping

The debate over the superiority of coiling or clipping has been extensively discussed, especially in the ruptured setting [20, 23, 28, 40, 46]. However, population-based outcomes are often challenging to apply to an individual patient under one’s care. We adopt a philosophy in which endovascular and open surgical interventions are complementary rather than competing techniques, and the values and shortcomings of each modality are carefully considered. A higher proportion of endovascular treatments may have been expected in this modern series. However, our report demonstrates that open microsurgical therapy was required in 38% of cases. This could be a reflection of the complexity of the aneurysms encountered in a tertiary referral center. The proportion of surgical cases may also reflect the training background and familiarity of the senior author for treating these aneurysms surgically, compared with a relative reluctance to trial less well-established endovascular strategies. Examples of such scenarios include instances where a need exists to catheterize very small perforator aneurysms or to stent-coil ruptured aneurysms.

The surgeon who performs both techniques must weigh the minimally invasive nature of endovascular therapy with the more definitive aneurysm obliteration afforded by microsurgery. When selecting the surgical option, the dual-trained surgeon should strive to achieve the safety profile and typical post-procedural recovery of the endovascular approach. When selecting the endovascular option, the rate of permanent complete occlusion achievable with open surgery should be the goal. This friendly competition between modalities encourages continual improvement and ensures that the practice pattern of a dual-trained surgeon remains grounded in standards of care of the wider cerebrovascular communities.

**Fig. 6:**
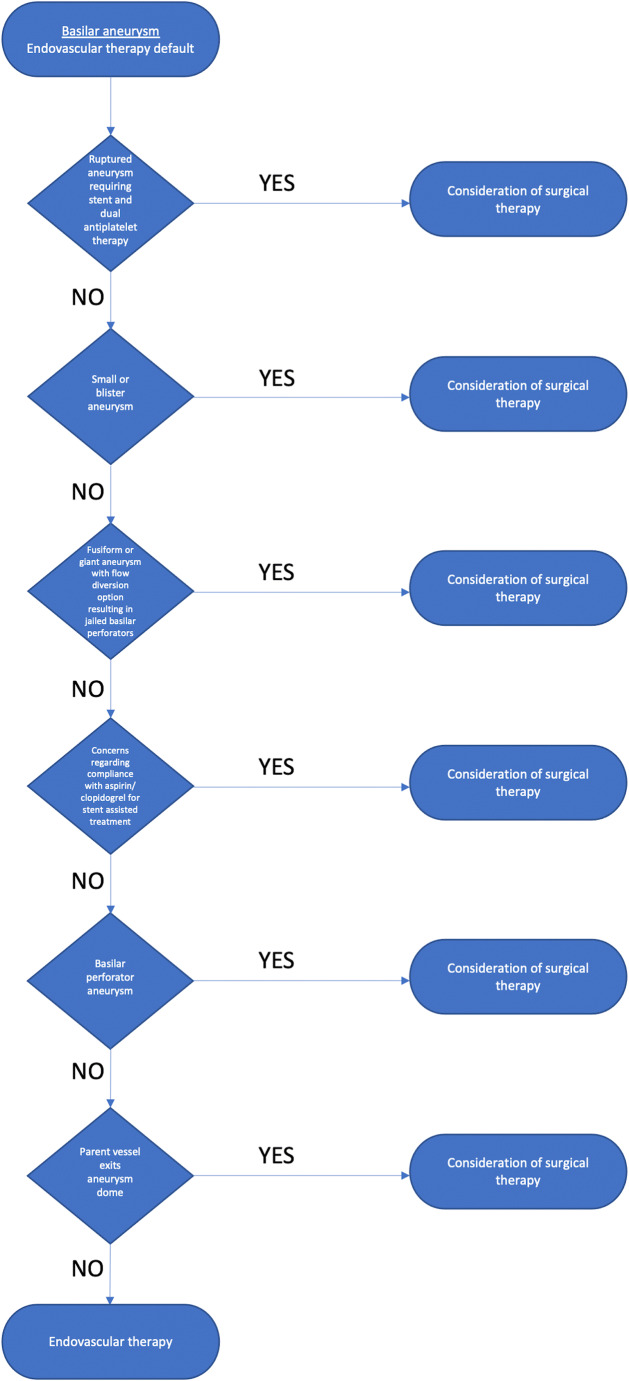
Suggested decision-making algorithm. A suggested decision making algorithm for basilar artery aneurysms is depicted in the flow diagram, based on the aneurysms and patients encountered and treated in this series

Multiple studies have supported superior clinical outcomes after treatment with endovascular coiling for ruptured aneurysms—especially in the posterior circulation [20, 23, 28, 40, 46]. In line with this evidence, we favor an endovascular first philosophy for BAAs in the ruptured setting. If the endovascular option is not possible or has a high risk of morbidity, careful consideration is given to surgical clipping. Others have described aneurysm morphological parameters which favor coiling or clipping, such as dome-to-neck ratios or neck diameters [13, 26, 39]. With the evolution of an expanded repertoire of endovascular treatments, various wide-necked aneurysms may be safely treated endovascularly [5, 26]. The majority of the aneurysms treated in this clinical series were wide-necked (neck diameter ≥ 4 mm) [13], and there was a trend towards more wide-neck aneurysms in the surgical cohort. Either eloquent vessels arising from the aneurysm dome or aneurysms arising from basilar perforators were the leading morphologic considerations favoring clipping in this series. Basilar perforator aneurysms are particularly challenging to treat endovascularly, as they are often small (<2 mm) and present an increased risk of intraprocedural rupture during catheterization, as well as an increased risk of perforator thrombosis after treatment. For ruptured wide-neck aneurysms that would require stent assistance to treat the aneurysm endovascularly, we favor clipping to avoid morbidity associated with dual anti-platelet medications in the ruptured setting [2, 6, 30]. Based on the aneurysms and patients encountered and treated in this series, a suggested decision-making algorithm for basilar artery aneurysms is depicted in Fig. 6.

An important consideration in selecting a treatment for BAA, especially in the unruptured setting, is to evaluate its ability to achieve complete and durable exclusion of the aneurysm from the circulation. Clipping has been shown to have greater durability with lower rates of recurrence and re-treatment compared with endovascular therapy [20, 23, 28, 41]. This is reflected in our outcomes (Table 3). Prior studies—such as the Cerebral Aneurysm Rerupture After Treatment trial—suggest that degree of aneurysm occlusion is a strong predictor of post-treatment hemorrhage [17]. For unruptured aneurysms, rates of bleeding from small neck remnants are much lower (< 1%), but may be elevated among posterior circulation aneurysms [29]. A recent meta-analysis demonstrated that incomplete occlusion is associated with higher rates of re-treatment [7]. The morbidity of re-treatment is additive, and the psychosocial stress of repeated surveillance and/or treatments has not been well investigated. With longer follow-up, clinical outcomes for posterior circulation aneurysms approach equivalence regardless of treatment approach [41].

**Table 3:** Summary of endovascular and open case series for treatment of basilar artery aneurysms

Endovascular					
Series	Year	Number	Complete occlusion (%)	Good outcome (%)^**^	Mortality (%)
Limbucci	2010–2013	52	88	-	2
Jeon	2008–2012	25	44	88	0
Chalouhi	2004–2011	235	88	-	0
Spiotta	2002–2010	19	26	-	4
Kulcsar	2008–2009	12	17	75	0
Henkes	1992–2005	316	58	77	1
Pandey^1^	1995–2005	275	88	87	5
Lusseveld	1994–1999	44	73	89	4
Uda	1990–1999	39	85	90	3
Bavinzski	1992–1998	58	41	57	12
Gruber	1993–1996	21	66	95	0
Nichols	1992–1996	26	60^2^	81	15
Raymond	1992–1995	31	42	87	3
McDougall	1991–1995	33	21	-	12
Pierot	1992–1994	35	71	91	9
Gugliemi^1^	1990–1991	42	33	90	10
Stapleton	2007–2014	46	48	-	-
Open surgery					
Series	Year	Number	Complete occlusion (%)	Good outcome (%)^**^	Mortality (%)
Sekhar	2005–2012	37	92	76	8
Nanda	1992–2009	62	92	77	8
Lawton	1997–2006	162	98^*^	63	9
Samson	1978–1996	302	94	81	9
Krisht	1998–2006	51	98	90	4
Lawton	1997–2001	56	-	84	9

^*^Includes aneurysms of basilar artery and branch vessels (including PCA and SCA) and for those with angiographic data available

^**^Good outcome defined as Glasgow Outcome Scale (GOS) score of 4 or 5 modified Rankin scale (mRS) score of 0–2 depending on study

^1^Includes all posterior circulation aneurysms

^2^Earliest angiographic follow-up reported was 6 months

Our consideration of patient-specific factors is not limited to age and medical comorbidities. Psychiatric and psychosocial factors, such as the patient’s ability to attend follow-up and likelihood of compliance with medical therapies, are important considerations that have not been systematically reported. In patients who are unlikely to follow up for radiographic surveillance, clinic visits, or to comply with medical therapies, consideration of surgical clipping is warranted. One patient in this series was habitually using methamphetamine, and initially underwent surgical treatment due to concern for antiplatelet non-compliance. He did not return for follow-up for over 1 year, continued to use methamphetamine regularly, and his fusiform basilar apex aneurysm recurred (the only surgical reoperation in the series) and required additional treatments and more vigilant follow-up.

### Comparison with prior clinical series

To our knowledge, there is only one prior institutional series reporting combined endovascular and open surgical treatment for BAAs [39]. There are several important differences between these two studies: theirs included only basilar apex aneurysms; the treatment team comprised of multiple practitioners; and surgeons were more advanced in their careers. Nevertheless, our reported rates of aneurysm occlusion and favorable outcomes are consistent with results from other clinical series of BAAs treated by a single approach (Table 3) [1, 5, 10, 11, 14, 15, 18, 19, 21, 24, 25, 27, 31–35, 37–39, 42–44]. Our occlusion rates with clipping are slightly lower than prior series, and we attribute this to inclusion of fusiform aneurysms of the basilar trunk or apex (*n* =5) and potentially to the learning curve in an early career series. All incompletely occluded aneurysms after surgical clipping occurred in the senior author’s first 2 years of practice, supporting the observation that there is a learning curve for these complex surgical approaches [21].

Others have reported that practitioners may acquire sufficient expertise to perform both endovascular and open surgery with outcomes similar to those who specialize purely in one treatment modality [3, 4, 8]. Our results extend these results to basilar aneurysms, and support that technical proficiency may be achieved relatively early in one’s career. Additionally, evaluating a series of BAAs treated by a single operator demonstrates that there is still a need for open surgery in a significant proportion of cases.

### Limitations

The current study is a retrospective analysis of a non-randomized consecutive case series of patients undergoing endovascular or open surgical treatment of BAAs. Although baseline demographics did not statistically differ between the groups, treatment allocation, as described above, may influence the direct comparability between endovascular and open surgical groups. Our study reflects a single surgeon experience, with patients treated at academic medical centers that function as tertiary referral centers for cerebrovascular pathology, and was limited to the first 5 years of clinical practice.

## Conclusion

The complementary use of endovascular and open microsurgical treatment of BAAs demonstrates non-inferiority of outcomes following clipping when compared to coil embolization. Good neurologic outcome was achieved in 88.5% and 75.0% of patients in endovascular and open cohorts, respectively (*p* = 0.40). Univariate analysis demonstrated that advanced age (OR 1.11 [95% CI 1.01–1.23]) or peri-procedural adverse event (OR 85.0 [95% CI 6.5–118.9]), but not treatment modality (OR 0.39[95% CI 0.08-–2.04]), were predictors of poor neurologic outcome.

Complementary implementation of both endovascular and open surgery facilitates individualized treatment planning for BAAs. By evaluating BAAs for treatment via both endovascular and open modalities and leveraging the strengths of both techniques, equivalent clinical outcomes and technical proficiency may be achieved with both modalities.
